# Artificial intelligence and robotisation in the EU - should we change OHS law?

**DOI:** 10.1186/s12995-021-00301-7

**Published:** 2021-05-05

**Authors:** Maciej Jarota

**Affiliations:** Department of Labour Law and Social Security Law, Institute of Law, John Paul II Catholic University of Lublin, Al. Raclawickie 14, 20-950 Lublin, Poland

**Keywords:** Employee health, Artificial intelligence, Employer, European Union, European legislation

## Abstract

**Background:**

Technological progress in the twenty-first century offers real chances for economic development of the European Union (EU). The purpose of this publication is to analyse risks and threats relating to Occupational Health and Safety (OHS) considerations in the context of scientific and technological development. The article attempts the analysis of whether current legislation of the European Union enables good protection of workers’ health in the performance of their duties using robots, artificial intelligence (AI). A feature of robotisation and AI may be new challenges in OHS protection. The analysis performed aims to determine whether threats posted by working with Artificial Intelligence are serious enough for the EU Legislator to focus on implementation of new OHS regulations.

**Methods:**

The analysis was carried out on the basis of current legal regulations related to the protection of employee’s health in the European Union. The study used literature related to robotisation with artificial intelligence and health and safety at work in the working environment.

**Results:**

Given the new psychological and physical threats related to the use of AI robots, it is necessary to expand the EU legislation with general guidelines for the use of intelligent robots in the work environment. Indeed, such robots must be defined in the applicable legal framework. Employers should also define, as part of their internal regulations, the procedures for employee communication with artificial intelligence, and relevantly update their training in the OHS area.

**Conclusions:**

The developments in AI-assisted robots come with inherent risks and threats to the working environment. New challenges create the need for adapting EU laws to changing reality. In order to structure European Union legislation on health and safety at work, these changes could be defined in a single piece of legislation covering robotics and AI after detailed analysis, dialogue, and debate.

## Background

Ever since the dawn of the first industrial revolution in the nineteenth century, the technology has developed to become a critical part of socio-economic life of European nations [1]. Socio-economic progress of European society improves the standard of life of all Europeans [2]. Presently the European Union sees the increase in new technologies contributing to organisation of employees’ work. Specific areas can be identified whose very existence is testimony to scientific and technological progress in the area of labour. Such areas include robotisation and AI. Their development may pose some risks, resulting in challenges to ensure adequate levels of safety and health protection in working environments.

Working with robots may involve risks posed by their physical use at the workplace. Incorrect operation or machine error may ultimately lead to undesirable effects, in particular to workplace accidents. First, due to certain level of autonomy of AI-assisted robots, inadequate communication between a robot and a human may cause robot behaviour, which is not controlled by a human, ultimately affecting the safety of workers. A new threat relating to the occurrence of AI-assisted robots at the workplace is the risk of aggravation to the mental health of workers. Workers may become stressed by the possibility of losing their jobs or the quality of their work being monitored by robots, which might lead to competition between human workers and robots [3, 4]. It is also easy to imagine workers’ frustration caused by the perceived ‘lesser’ quality of their work as compared with that of AI-assisted robots.

It begs the question of whether EU legislation requires implementing new legal solutions with regard to protection of workers’ health and safety. The article analyses the impact of robotisation, AI on the protection of worker’s health and safety, as those very areas introduce to the work environment new phenomena affecting occupational health and safety. The afore-mentioned areas have one thing in common - they are subject to EU legislation governing OHS regulations protecting workers, in particular Council Directive 89/391/EEC [5]. Further, areas subject to the study determine the directions of scientific and technological progress. This is why the article is focusing on matters related to the analysis of European legislation regarding occupational health and safety, in the context of challenges posed by development in the areas subject to the study. Such challenges should be sufficiently addressed both by EU Legislator and recipients of such legislation - first and foremost the employers. These research is the starting point for further discussion of the need for regulation of health and safety protection at the work place in the context of new scientific and technological challenges that work processes are facing.

### Robots and artificial intelligence - meaning and definition

Robots can be programmed to complete new tasks. The literature defines robots as re-programmable multi-purpose devices designed for the handling of materials and tools for the processing of parts or specialised devices by means of varying programmed movements in order to complete a variety of tasks. First robots appeared at the workplace in 1960s [6], although the word “robot” was coined as early as in 1920 [7]. According to the International Federation of Robotics (IFR), in 2016 there were 1.8 million industrial robots in operation [8]. The number of workplace robots certainly continuous to increase systematically [9].

The basic question is whether a robot may be a machine, or only software. The literature assumes that a robot is a physical object [10]. Robots may be divided into autonomous and non-autonomous. The former may make decisions independently, based on information acquired [11]. The latter are regular work tools, acting on a pre-programmed algorithms created by a robot’s owner. Autonomous robots maybe programmed by means of so-called “machine learning” which may be defined in a variety of ways; also as a learning process consisting in computers not only acting but also learning like humans, perfecting their learning process in an autonomous manner, by providing them with data and information in the form of observations and real life interactions [12]. By another definition, machine learning is the learning process consisting in computers acting without any specific programming [13]. The European Parliament stressed that machine learning is the component of Artificial Intelligence (AI) that ensures the possibility of automatic learning on huge volumes of data, whereas machine learning algorithm may be perceived as “one algorithm generating another algorithm” – model [14]. There are three types of machine learning: supervised (based on data marked for mode generation), unsupervised (absence of marked data, automatic identification of pattern and structure from training data) and enhanced learning based on the use of feedback from success and failure received from the environment [15].

Artificial Intelligence is defined in a number of ways. One of the definitions specifies that it is a branch of IT dealing with simulated intelligent behaviour of computers or machines simulating the intelligent behaviour of humans [16]. AI is based on the ability to perceive specific environment and activities performed therein by means of processing digital data [17]. The combination of smart algorithms and large quantities of fast processed data enable automatic learning on the basis of data models and features. By using suitable technology, AI may process, analyse and understand images, capture still and moving images and interpret their ambience, all in real time. In the consequence of all cognitive actions, AI is able to learn, understand and perform specific tasks using information provided to it [18]. In the European Parliament it was noted that narrow (weak) AI is designed to perform specific tasks, such as identification of faces or product recommendations, whereas general (strong) AI is designed to outsmart people in a number of disciplines [15]. AI-assisted robots may have self-awareness [15]. In the Resolution of 16 February 2017 with recommendations to the Commission on Civil Law Rules on Robotics (2015/2103(INL)), the European Parliament called the Commission to postulate a common EU-level definition of cyber-physical systems, autonomous systems, intelligent autonomous robots and their sub-categories, and defined the features of intelligent robots: acquiring autonomy by means of sensors or exchanging data from the environment (reciprocal connections), including exchange and analysis of such data; the ability to learn from the experiences gathered and interactions with the environment (optional criterion); at least the minimum physical form; matching of behaviour and actions with the environment; absence of life functions in a biological sense [6].

Presently, AI is used i.e. in the construction of autonomous vehicle systems. The example of such autonomous vehicle may be Google’s smart car [18]. AI applications also include medicine [19–21]. Robots utilising AI already complete tasks of some professionals, e.g. delivery of food [10]. Hospitals utilise AI in surgery [19], among others, and banking sector utilises AI in customer service operations [18].

The European Commission maintains that the use of AI is a strategic and critical factor of economic development. AI has to serve both the European Union’s society and economy [18]. An ever increasing number of robots utilising AI, already referred to as “electronic persons” in the European Union enables improvement in work efficiency [22].

## Methods

### Purpose of the study

The purpose of this publication is to analyse risks and threats relating to OHS considerations in the context of scientific and technological development. The analysis performed aims to determine whether threats posted by working with Artificial Intelligence are serious enough for the EU Legislator to focus on implementation of new OHS regulations. The study attempts the analysis of whether current legislation of the European Union enables good protection of workers’ health in the performance of their duties using robots, AI.

### Material and analysis

The analysis made use of literature related to robotisation using AI. Not only were the achievements of legal sciences used, but also publications dealing with occupational health and safety. The research was carried out on the basis of the current legal regulations related to the protection of workers’ health in the European Union. The publications used were verified in terms of methodology and qualified on the basis of the convergence with the subject. The literature review was based particularly on the following databases and online journals: HeinOnline, PubMed, Scopus, Web of Science, ERIH PLUS, and EBSCO. Sources were identified via keyword-based searches in such databases, as well as in online repositories and digital libraries, taking into account the publication date and author, and the paper type (scientific, popular science). The selection of literature was based on detailed analysis of specific texts in terms of individual research topics. Following the review, numerous publications were excluded because their subject turned out to be irrelevant. On the other hand, the final selection included research papers that were considered significant for answering the research questions asked in this paper. Apart from analysing the texts sharing the same perspective, the review also covered the sources presenting different positions. When working with robots equipped with artificial intelligence, the topic of health protection has not yet been comprehensively described in the literature. Nevertheless, selected publications have made it possible to identify certain directions of research on the use of AI robots in the work environment from the perspective of employee health protection.

## Results

### Major legislative areas of the European Union in occupational health and safety

In the basic legal act on OSH, i.e., Council Directive 89/391/EEC, the employer is obliged to take the measures necessary for the safety and health protection of workers, including prevention of occupational risks and provision of information and training, as well as provision of the necessary organisation and means. Article 6(1) of the Directive provides for the employer’s obligation to adjust these measures to take account of changing circumstances and aim to improve existing situations. Furthermore, Article 6(3) of the Directive introduces a rule that the employer must, in all planned undertakings, take into account the nature of the activities of the enterprise, the risks to the safety and health of workers at work in the choice of work equipment, the chemical substances or preparations used, and the fitting-out of workplaces. Subsequent to this evaluation and as necessary, the preventive measures and the working and production methods implemented by the employer must, in the first place, assure an improvement in the level of protection afforded to workers with regard to safety and health. According to Article 9(1) of the Directive, the employer should be in possession of an assessment of the risks to safety and health at work, including those facing groups of workers exposed to particular risks, and decide on the protective measures to be taken and, if necessary, the protective equipment to be used. The employer should keep a list of occupational accidents resulting in a worker being unfit for work for more than three working days and draw up, for the responsible authorities and in accordance with national laws and/or practices, reports on occupational accidents suffered by its workers (Article 9(1) of the Directive). The basic instrument of occupational health and safety at work is risk assessment in the workplace. In the context of scientific and technological advancements, risk may take various forms.

Referring to the use of robots in a work place, there are acts of law in the European Union also relating to the safety of workplace equipment. In particular, Council Directive 85/374/EEC [23] (with included the principle of liability for movables, regarding the liability of any manufacturer for defective products, should it fail to ensure such safety as a person is entitled to expect), and Directive 2001/95/EC [24]. The Directive 2001/95/EnC defined requirements to be met by a product to be considered as safe, and its Article 11 set out the principles for Member States notifying the implementation of measures restricting the marketing of products or mandating their withdrawal.

Human-machine interactions are governed by Directive 2006/42/EC [25] which implemented mostly general OHS requirements with regard to design, execution and operation of machinery and equipment. The Directive sets out rules for constructing operation manuals for machinery and equipment, and imposes the obligation on a manufacturer to perform the risk assessment. As defined in Art. 2(a), the “machine” is an assembly, fitted out with or designed to be fitted out with a drive mechanism other than human or animal muscles, comprising interconnected parts or components of which at least one is moving, combined together for specific application. Also, as defined in Article 3 of the Directive, prior to marketing or commissioning of the machine, the manufacturer or his or her authorised representative ensures, e.g. that the machine meets relevant general OHS requirements, as detailed in Appendix I. The contemplated appendix defines in particular principles of controlling the machine which do not consider the aspect of autonomous control and in particular its self-awareness.

In European Union Directive 89/391/EC defines general OHS principles applicable in every work environment utilising intelligent robots. Relevant principles with regard to machines have been provided for, also in Directive 2006/42/EC. The fact of regulating OHS matters on an EU level has to be appraised as positive. From this perspective it is vital to determine whether present EU-level legislation is considering threats and risks posed by robotisation, and artificial intelligence. EU regulations do not govern detailed new aspects of labour processes related to advancements in robotisation.

The EU legislation was created in different scientific and technological reality. No principles for using AI-assisted robots considering the specificity of AI can be found there. There are no regulations governing controlling of robots with self-awareness. The definition of machine included in Directive 2006/42/EC was created in the reality with state of the art much different from today’s. It does not define levels of autonomy for robots which in the future may play a significant role in the work process of many European employers. This makes the analysis of risks and threats posed by new areas of scientific and technological progress a justified task.

Recently, attention has been drawn in the European Union to principles for robot use. It is reflected in the European Commission postulating in 2018 the creation of a European framework for AI functioning. In the same year the agreement relating to AI was executed by 24 Member States and Norway [17]. In addition, the European Agency for Safety and Health at Work (EU-OSHA) indicated AI assisted tools and applications functioning at the workplace, presenting the consequences of their use. Subject to analysis in the EU were e.g. decision-making applications for a workplace, indentifying related risks and recommendations concerning risk management measures [25]. Also, EU level debate may be observed in which the regulation of robots’ legal status is postulated. The European Parliament proposes definition and taxation of robots, and specification of their obligations [17].

There is no doubt that the Commission has been involved for the last three years in studying the area of AI, by issuing communications [26, 27]. In addition, independent experts from the European Commission have made general recommendations on the development of AI [28]. The Commission’s recent activities include an open public consultation on artificial intelligence in relation to the White Paper on Artificial Intelligence – A European approach to excellence and trust published on 19 February 2020 [29]. Despite the European Commission’s activities, employers undertake their own initiatives, developing in-house principles for the use of robots at work. Unfortunately, they are rarely generally available for use by other employers [25].

### Main threats and risks relating to robotisation and artificial intelligence at the workplace

As indicated above, robotisation and AI may affect working conditions, and thus their occurrence should be subject to the analysis in the context of ensuring safe and healthy working conditions to workers. Despite a number of regulations applicable on European Union level one needs to consider the need for a review of the existing legislation governing occupational health and safety. The literature also points out future challenges, stemming from the use of artificial intelligence in work environment [25]. The issue consists in whether the legislation applies to all areas of worker health and safety protection at work places utilising robots [30].

Robots may, in turn, operate in a complementary manner at the production hall, assisting human workers. The aforementioned may, however, be connected with H&S - related threats, in particular damage or collision between a human and a robot, due to defective sensors, software or connectivity [25]. When analysing the problem of artificial intelligence, it has to be observed that when faced with delegating some employee duties onto an AI robot, a few issues emerge relating to understanding the principles governing its work at the workplace. Firstly, how the communication between a robot and a human is to be effected [4, 30]. Secondly, if some duties are to be performed by a robot (which might interact with human, including making decisions on behalf of the employer), will such robot perform functions entrusted to it considering specific ethical principles [4, 31]. As opposed to a human, a robot is deprived of any emotions [4, 5, 32].

Development of AI generates new human and machine interactions, improves the performance, but also poses threats to privacy of employee and his or her protection against discrimination [33]. Employees may be however most concerned about losing their jobs, hence the risk of future competition for jobs between humans and robots [2], which might indirectly affect occupational health and safety [4, 34].

Scientific research has been conducted on relationships between humans and artificial intelligence, which focuses on improving the perception of robots [35]. There is no doubt about the possibility of humans working near robots, provided the workplace is well organised [36]. It is however difficult to predict accurately all risk factors in protection of workers’ health [4, 37, 38], especially regarding interactions between humans and robots with self-awareness. Therefore, the only known relationship between human and machine, where a human operates and controls the machine, or completes a task using a machine, is no longer the only scenario for the use of a machine in a workplace. Intelligent robots involved in the process may make their own decisions, based on their own experience and interactions with the environment. AI-assisted robots involved in the work process and work organisation, may pose new workplace risks that are not yet known or identified. It is even projected that in the future a number of fatal accidents involving robots shall increase due to expanding robotisation of companies [34]. On the other hand, keep in mind the positive effect of new technology on the workplace OHS. Algorithms occur that instead of actual work of employee enable managers assessing workplace safety using software by identification of risks [39].

Considering employee-robot interaction, the question arises whom shall be liable for damage caused by the AI. It seems that actions taken by robots, which sometimes decide on behalf of employers, cause material implications at work. The literature stresses the need for considering whether some legal framework needs to be implemented governing the risks posed by robot actions, including the scope of liability for incidents caused by certain actions of robots [40].

## Discussion

How then, to evaluate European OHS legislation in the context of consequences of work with AI? The EU legislation also fails to define artificial intelligence, although such facts as the afore-mentioned resolution of the European Parliament of 16th February 2017 monitoring to regulate this area, are positive. Directive 2006/42/EC applicable to machinery failed to introduce a universal definition of AI-assisted robots having even partial self-awareness. The Directive may be useful when ensuring OHS at the workplace utilising traditional machinery. It is applicable to the principles of machine operation, without considering the impact of artificial intelligence that in certain situations may be able to make decisions independently. The above analysis demonstrates that European Union legislation does not address some new threats relating to the protection of employees’ health. In fact, EU legislator generally regulated the matters of OHS, in particular in Council Directive 89/391/EEC; however, legal standards are missing that refer directly to threats and risks relating to AI at the place of work. In particular, the issue consists in the absence of regulations governing controlling of robots with self-awareness when there are risks associated with human-robot interaction.

Ever more common use of robots at the workplace may pose more risks - especially to new forms of robots’ use. Performance of workers’ duties should be so organised as to guarantee them the availability of protective measures preventing accidents at work. Workers should be adequately instructed in the observation of general OHS principles at work. Work using AI-assisted robots should be based on clearly defined principles of human-robot interactions, so as to avoid any misunderstandings with regard to specific tasks performed at work.

The new risks associated with robotisation, AI in the workplace make it necessary for the EU legislator to reconsider the current rules. On the other hand, a robot equipped with artificial intelligence has a particular impact on another area of health and safety that concerns the relationship between a human being and such a robot performing certain tasks in the workplace. It is not limited to the worker’s contact with any substance, but concerns a mechanism for using artificial intelligence in the work process that can cause both psychological and communication risks.

European legislation refers to OHS risks and threats, clearly indicating employers’ obligations to provide workers with adequate work conditions. Employers are often forced to define procedures mandatory to workers, by implementing in-house regulations, in order to ensure health and safety in the workplace. Obviously, no legislation should substitute the good OHS practices of employers, who should consider a host of risks posed by new technologies. Comprehensive EU legislation points out potential risks and threats to the recipients of legal standards. Therefore, it should be considered if any legislative changes on a EU-level have to apply to all above-mentioned aspects missing regulations, or if it would be sufficient to leave regulation of workplace OHS to employers, to some extent.

The scale of risks analysed requires in-depth reflection. There are two ways to do it - a hard law is implemented, in the form of an EU directive or regulation, or a soft law is implemented in the form of recommendations and leaving the day-to-day management of OHS matters to stakeholders [41, 42].

On the one hand the smallest possible intervention of the European Union is postulated. Such soft laws do not impose ready-made solutions, but only initiate undertakings by employers of their own actions, taken in their own best interest, notifying them of specific threats. In this context soft legal tools may ensure adequate protection of employees’ health at the place of work, especially when they are formulated in dialogue with personnel [43]. Autonomous regulation of employee protection measures, considering specificity of each place of work, may have better result in each case than hard laws [44]. OHS procedures established by an employer should consider workplace specifics, in particular adequate metrics for the adopted labour culture [45]. Each employer is required to act in a specific way not only due to laws but also due to Corporate Social Responsibility [46].

On the other hand an argument may be raised that adoption of soft laws to define the level of health protection is insufficient. In the absence of binding regulations, recommendations issued by the European Commission may result in OHS procedures being adopted by only the small number of employers [47]. In addition, various cultures and methods of governance by legal institutions in Member States using soft laws lead to different results of legislative activities. The consequence of individual legislative actions may be not uniform level of OHS in the European Union work environment [48].

Any private initiatives may be of an auxiliary nature, it should be critical to define laws based on scientific research and adequately addressing the existing state of OHS. It seems, therefore, those recommendations or other soft forms of law that do not impose binding obligations on Member States, will not play a significant role in implementation of safety procedures and measures [47]. The starting point when formulating any legislative proposals shall be the purpose of law [49].

That the need for regulation of this issue in the European Union law in the form of a directive or regulation is justified is evidenced by European experience in the implementation of OHS laws. Obviously, broadly understood healthcare of EU citizens requires, in general, a comprehensive approach of European Union, and not only by ensuring observation of workplace OHS [50]. The European Commission published on 5th February 2004 [51] the communication on practical implementation of OHS regulations, i.e. Directive 89/391/EEC, indicating in particular, based on national reports, the positive outcome of European Union legislation on national OHS standards. First and foremost, in the Commission’s opinion, they helped alleviate workplace risk and increased the awareness of European society with regard to existing obligations aiming to ensure adequate working conditions.

When analysing threats related to the use of AI-assisted robots in work processes, the conclusion is justified that general OHS principles stemming from the Directive 2006/42/EC are not sufficient. As regards work involving the use of AI-assisted robot it is difficult to implement uniform principles of hard laws due to the rapidly changing technical state of the art. Certainly *de lege ferenda* AI-assisted robots have to be defined based on hard laws. Implementation of general european procedures for workplace use of such robots is also worth considering. Their independence, especially the potential for partial or full self-awareness requires answering the question how to treat a robot at the workplace – in the same way as a human? The problem of charging AI-assisted robots with specific OHS duties, and then enforcing such obligations from them, is questionable. Under present legislative reality it is not possible, since a robot is not a worker. This problem certainly requires additional research. It seems that implementation of general guidelines in hard laws at the EU level with regard to principles of using intelligent robots in the work process would be helpful to employers. Considering the needs of their plans and tasks assigned to robots, employers should define principles of employees’ communicating with AI on their own, as accurately as possible, under in-house regulations, taking into account also possible updates to OHS training of personnel. At this stage the employers should obligate the workers to exercise extreme caution when interacting with intelligent robot.

Summary there is no single piece of legislation that would directly and comprehensively cover new workplace scenarios related to AI. The general EU Directive 89/391/EC and Directive 2006/42/EC lay down only basic health and safety standards of a general nature. There is a need to implement specific legal solutions at the European Union level, when defining AI considering its potential level of self-awareness in the work process.

## Conclusions

To sum up, these studies indicate that AI-assisted robots’ development comes with inherent risks and threats to the working environment. Such risks and threats are physical and psychological in nature. Their occurrence should motivate further debate and pursuing the answer about the extent to which EU-level OHS regulations need to be implemented. New challenges create the need for adapting EU laws to changing reality. EU-level standards should consider new circumstances referring especially to advancements in AI in work processes. Detailed scope of possible legislative changes surely requires in-depth analysis, dialogue and debate, so as to enable precise and effective addressing of threats occurring in the work place. In order to structure European Union legislation on health and safety at work, these changes could be defined in a single piece of legislation covering robotics with AI.

In the context of AI, soft law must not be forgotten. Some threats may undoubtedly be eliminated with soft laws. Employers themselves can take concrete steps to ensure that their employees have adequate working conditions, without the EU legislator imposing ready-made solutions on them.

## Data Availability

Not applicable.

## Abbreviations

AI: Artificial intelligence
OHS: Occupational Health and Safety
EU: European Union
